# Perceived Pain during Cataract Surgery with Topical Anesthesia: A Comparison between First-Eye and Second-Eye Surgery

**DOI:** 10.1155/2015/383456

**Published:** 2015-05-04

**Authors:** Lin Jiang, Keke Zhang, Wenwen He, Xiangjia Zhu, Peng Zhou, Yi Lu

**Affiliations:** ^1^Department of Ophthalmology, Eye and Ear, Nose, and Throat Hospital, Fudan University, 83 FenYang Road, Shanghai 200031, China; ^2^Key Laboratory of Myopia, Ministry of Health, 83 FenYang Road, Shanghai 200031, China; ^3^Shanghai Key Laboratory of Visual Impairment and Restoration, Fudan University, 83 FenYang Road, Shanghai 200031, China; ^4^Department of Ophthalmology, Parkway Health, Specialty and Inpatient Center (Luwan), 170 DanShui Road, Floor 3, Shanghai 200020, China; ^5^Hong Qiao Medical Center, 2258 HongQiao Road, Shanghai 200033, China

## Abstract

*Purpose*. To compare pain scores between first-eye and second-eye cataract surgery and to determine the affecting factors. *Methods*. 106 first-eye and 53 second-eye cataract surgery patients (mean age: 67 ± 13 and 69 ± 10 years, resp.) were enrolled. The patients completed simplified State-Trait Anxiety Inventory and visual analog scale (VAS) for anxiety questionnaires before surgery, and VAS for pain and Wong-Baker Faces Pain Rating Scale questionnaires after surgery. Blood pressure (BP) and heart rate (HR) were recorded perioperatively. *Results*. A greater proportion of patients who underwent second-eye surgery reported intraoperative pain compared with first-eye surgery patients (85% versus 35%, *P* < 0.001). The pain scores were higher in second-eye surgery, while the VAS anxiety score was lower in second-eye surgery. Moreover, 31 patients reported greater pain during second-eye surgery than their first one, with higher pain scores than other 22 patients (*P* = 0.032 and 0.003, resp.). The VAS pain score of these 31 patients was positively correlated with the differences between the intraoperative and postoperative diastolic BP, mean arterial pressure, and HR. *Conclusions*. Cataract patients were likely to have more pain during second-eye surgery, which may be related to lower preoperative anxiety. Monitoring perioperative BP and HR may help to identify patients with intraoperative pain.

## 1. Introduction

Uncomplicated cataract extraction is usually conducted under topical anesthesia. Perioperative pain management not only reduces the patient's anxiety before and after cataract surgery, but also improves the patient's intraoperative cooperation. Therefore, pain management is particularly important when performing cataract surgery.

Currently, phacoemulsification plus implantation of an intraocular lens under topical anesthesia is the main surgical approach to treat cataract. Topical anesthesia significantly reduces the perceived pain at the time of making the clear corneal incision and small incision, as compared with historical techniques [1].

Previous studies, including our clinical practice, have revealed that patients experience more painful sensations during second-eye surgery. Although an earlier study found no significant difference in the mean pain scores between patients undergoing second cataract extraction compared with patients undergoing first cataract extraction [2], in 2011, Ursea et al. reported for the first time that there was a subtle increase in pain during second-eye surgery compared with first-eye surgery [1]. Tan et al. also found that the pain during second-eye surgery was significant even on multivariate analysis [3].

Several studies have examined the possible causes of the increased pain during second-eye surgery. For example, Ang et al. found that nearly 20% of patients reported frightening intraoperative visual experiences that were associated with previous cataract extraction [4]. However, factors have been proposed. Nijkamp et al. reported that previous cataract extraction was weakly, but negatively, correlated with preoperative anxiety [5]. Boker et al. reported that the mean fear and anxiety scores were not significantly different between first-eye and second-eye cataract surgery [6]. However, earlier studies mainly focused on the patient's subjectively evaluated anxiety, and the results may be influenced by bias due to individual differences in pain threshold and their comprehension of the questionnaires used. Perioperative blood pressure and heart rate were objectively measured as possible markers of the patients' anxiety levels.

Therefore, we used subjective and objective measures in the perioperative period, with the following aims: (1) to compare the anxiety and pain scores between first-eye and second-eye cataract surgery, (2) to identify factors correlated with the severity of pain during cataract surgery, and (3) to help surgeons evaluate and manage perceived pain during cataract surgery.

## 2. Materials and Methods

### 2.1. Patient Collection

The Ethics Committee of the Eye and Ear, Nose, and Throat Hospital, Fudan University, approved our study. Patients with bilateral age-related cataract were considered eligible for this study. Exclusion criteria included baseline eye pain, deafness, poor compliance to cataract surgery under tropical anesthesia, involuntary movement, history of allergy to topical anesthetics, posterior capsule organization, or other complicated cataracts. Patients were enrolled between April 2013 and July 2013. Written informed consent was obtained from all patients after they were informed of the nature and possible consequences of the study. The consent procedure was approved by the hospital's ethics committee.

### 2.2. Subjective Scales and Questionnaires

Preoperative anxiety was evaluated using the validated simplified State-Trait Anxiety Inventory (STAI; 6 questions) [7], and a visual analog scale (VAS) for anxiety, which was presented as a numbered line ranging from 0 (no anxiety) to 10 (unbearable anxiety) [8]. The English and Chinese versions of questionnaires on anxiety and pain evaluation were provided in Supplementary Material available online at http://dx.doi.org/10.1155/2015/383456.

Postoperative pain was evaluated using a VAS for pain, which was presented as a numbered line ranging from 0 (no pain) to 10 (unbearable pain) [8] and the Wong-Baker FACES Pain Rating Scale (WBS), which comprised 6 faces ranging from a happy face for no pain (score = 0) to a crying face for worst pain (score = 10) [9]. Patients undergoing second-eye surgery were also asked to compare the severity of pain during their first-eye and second-eye surgery, with the following possible responses: “I had more pain during the first procedure,” “I had more pain during the second procedure,” “I experienced the same pain during both procedures,” or “I cannot remember.”

The preoperative anxiety assessments were completed while the patient was in the waiting room before surgery. The assessments were orally administered by a trained investigator. The postoperative pain assessments were completed when the patient was transferred to the recovery room. The questionnaires were administered by the same investigator. Patients who were unable to read the VAS for pain by themselves were asked to verbally report the perceived pain using the same scale.

### 2.3. Objective Measures

Systolic blood pressure (SBP), diastolic blood pressure (DBP), and heart rate were measured using an electric sphygmomanometer (HEM-907, OMRON, Kyoto, Japan) by an experienced nurse at the following times: before surgery, during phacoemulsification, and in the recovery room. Mean arterial pressure (MAP) was calculated using the following formula: MAP = SBP × 1/3 + DBP × 2/3.

### 2.4. Surgical Technique

The preoperative examination and surgery were strictly performed according to established outpatient surgical procedures in all patients. The same operating room was used for all procedures with the same surgical equipment and instruments. Tropicamide was administered 30 min before surgery to fully dilate the pupil. The conjunctival sac was rinsed with povidone iodine (0.02%) 5 min before surgery. Topical anesthesia consisted of 3 applications of 2% lidocaine before surgery, 1-2 drops per time, with the first application 5 min before surgery, the second application at 1 min before surgery, and the final application after placing the eyelid retractor. Oral and intravenous sedatives or analgesics were not permitted. All procedures were performed by the same right-handed surgeon (Yi Lu).

After topical anesthesia, a 2.6 mm temporal clear corneal incision was created, followed by viscoelastic (DisCoVisc; Alcon Laboratories, Inc., Fort Worth, TX, USA) injection and 5.5 mm continuous curvilinear capsulorhexis. Hydrodissection, chopping, nucleus rotation, and phacoemulsification were then performed. A foldable intraocular lens (SN60WF; Alcon Laboratories, Inc.) was implanted using a dedicated injector. After aspiration of residual viscoelastic, the incision was hydrated with balanced salt solution and checked for water tightness.

### 2.5. Statistical Analysis

All statistical analyses were performed using SPSS version 13.0 (SPSS Inc., Chicago, IL, USA). Quantitative data are presented as the mean ± standard deviation. The *χ* ^2^ test was used to compare categorical variables. Comparisons of continuous variables between two groups were made using two-tailed Wilcoxon's rank-sum test for nonparametric variables and Student's *t*-test for parametric variables. The changes in blood pressure and heart rate over time were analyzed by one-way analysis of variance (ANOVA) followed by the least significant difference test to compare means between group. Spearman's correlation analysis was used to analyze the correlations between selected variables. A *P* value of <0.05 was considered statistically significant in all analyses.

## 3. Results

### 3.1. Baseline Characteristics of the Patients

Between 1 April 2013 and 30 July 2013, 167 ARC patients undergoing cataract surgery were enrolled in this study. These 167 patients were administered the questionnaires, of which 159 provided valid responses and were analyzed in this study. The valid patients were divided into two groups, as follows: 106 patients underwent first-eye surgery and 53 underwent second-eye surgery. There were no significant differences between the two groups in terms of age and proportions of males/females (Table 1).

### 3.2. Patients Undergoing First-Eye or Second-Eye Surgery

#### 3.2.1. Comparison of Pain Perception

A significantly greater proportion of patients who underwent second-eye surgery (46/53 patients, 85%) reported pain during cataract surgery compared with patients who underwent first-eye surgery (37/106 patients; 35%) (*χ* ^2^ test, *P* < 0.001; Table 2).

#### 3.2.2. Comparison of Subjective Measures

Regarding the subjective anxiety measures, the median anxiety scores were lower in patients who underwent second-eye surgery than in patients who underwent first-eye surgery, especially the VAS score for anxiety (Wilcoxon rank-sum test, *P* = 0.047; Table 2).

VAS scores for pain exceeding 0 were considered to indicate perceived pain during surgery. We found that significantly more patients who underwent second-eye surgery perceived pain during surgery than patients who underwent first-eye surgery (89.79% versus 34.91%, resp.; *P* < 0.001; Table 2). Moreover, the VAS and WBS pain scores were significantly greater in patients who underwent second-eye surgery (Wilcoxon rank-sum test, *P* = 0.001 and 0.003, resp.; Table 2).

#### 3.2.3. Comparison of Objective Measures

**Figure 1:**
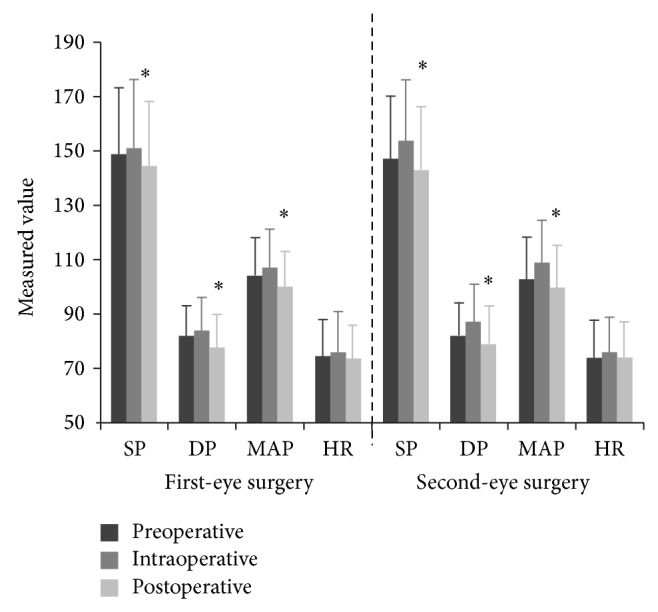
Comparison of blood pressure and heart rate among cataract patients who underwent first-eye or second-eye surgery. SP, systolic pressure; DP, diastolic pressure; MAP, mean arterial pressure; HR, heart rate. ^∗^Systolic blood pressure, diastolic blood pressure, and mean arterial pressure were significantly different between the postoperative and intraoperative measurements (all *P* < 0.05; one-way analysis of variance, followed by the least significant difference test).

Regarding the objective measures, there were no significant differences between the two groups in terms of the type of cataract, mean operating room time, or phacoemulsification time. Furthermore, there were no significant differences in perioperative blood pressure or heart rate between the two groups of patients (Figure 1). However, analysis of variance followed by the least significant difference test showed that SBP, DBP, and MAP were significantly lower after surgery than before surgery in patients undergoing first-eye surgery (*P* = 0.041, *P* < 0.001, and *P* = 0.001, resp.) and in patients undergoing second-eye surgery (*P* = 0.002, *P* = 0.003, and *P* = 0.004, resp.). The change in heart rate during surgery was not significantly different between the two groups of patients.

#### 3.2.4. Correlations between Objective and Subjective Measures

We next analyzed the correlations between the perioperative changes in objective measures (blood pressure and heart rate) and the subjective measures (preoperative anxiety and postoperative pain).

The preoperative VAS anxiety score was significantly and negatively correlated with the postoperative WBS pain score (Spearman's *ρ* = −0.300, *P* = 0.029).

Among patients who underwent first-eye surgery, the STAI and VAS anxiety scores were significantly and positively correlated with preoperative heart rate (STAI: Spearman's *ρ* = 0.257, *P* = 0.009; VAS anxiety: Spearman's *ρ* = 0.231, *P* = 0.002).

Among patients who underwent second-eye surgery, the VAS anxiety score was significantly and positively correlated with preoperative SBP (Spearman's *ρ* = 0.397, *P* = 0.003), DBP (Spearman's *ρ* = 0.278, *P* = 0.044), and MAP (Spearman's *ρ* = 0.349, *P* = 0.010). The STAI score was significantly and negatively correlated with the difference between intraoperative and preoperative MAP (Spearman's *ρ* = −0.300, *P* = 0.029).

### 3.3. Patients Undergoing Bilateral Sequential Cataract Surgery

#### 3.3.1. Changes in Perceived Pain

Patients who underwent second-eye surgery (*n* = 53) were divided into two subgroups according to their responses to the additional question regarding the difference in pain between the first and second surgeries (Table 3). Overall, 31 patients (58%) reported more severe pain in the second-eye surgery than in the first-eye surgery, while 22 patients (42%) reported similar or less-severe pain during the second-eye surgery.

#### 3.3.2. Changes in Subjective Measures

The subgroup of patients who perceived the second-eye surgery to be more painful also reported significantly greater VAS and WBS pain scores compared with the other subgroup (Wilcoxon rank-sum test, *P* = 0.032 and *P* = 0.003, resp.; Table 3). Age, proportions of males/females, preoperative anxiety score, type of cataract, mean operating room time, mean phacoemulsification time, blood pressure, and heart rate were not significantly different between these subgroups of patients. However, the median STAI and VAS anxiety scores were lower in patients who reported more severe pain during the second-eye surgery (Table 3).

#### 3.3.3. Changes in Objective Measures

There were no significant differences in perioperative blood pressure or heart rate between the two subgroups of patients (Table 3). Meanwhile, the changes in blood pressure and heart rate during surgery were not significantly different between the two subgroups of patients.

#### 3.3.4. Correlations between Subjective and Objective Measures

In the subgroup of patients who reported that second-eye surgery was more painful than first-eye surgery, the VAS anxiety score was significantly and positively correlated with preoperative SBP (Spearman *ρ* = 0.389, *P* = 0.031) and the STAI anxiety score was significantly and negatively correlated with the differences between the intraoperative and preoperative values for DBP (Spearman's *ρ* = −0.369, *P* = 0.041), MAP (Spearman's *ρ* = −0.451, *P* = 0.011), and heart rate (Spearman's *ρ* = −0.430, *P* = 0.016).

In the subgroup of patients who reported that second-eye surgery was more painful than first-eye surgery, the VAS pain score was significantly and positively correlated with differences between intraoperative and preoperative values for DBP (Spearman's *ρ* = 0.356, *P* = 0.049), MAP (Spearman's *ρ* = 0.371, *P* = 0.040), and heart rate (Spearman's *ρ* = 0.430, *P* = 0.016).

## 4. Discussion

For many years, cataract surgery was mainly performed under retrobulbar and nerve-block anaesthesia. Now, most of these ophthalmic procedures are carried out under topical anesthesia. This change in the anesthetic method is clinically significant because topical anesthesia reduces the rate of postoperative complications and reduces the postoperative rehabilitation time. However, patients may experience greater anxiety and pain during surgery. Our study revealed that a significantly greater proportion of patients undergoing second-eye surgery reported surgical pain compared with patients undergoing first-eye surgery, and the former group of patients also reported significantly greater VAS and Wong-Baker pain scores. Because there were no significant differences in age, proportions of males/females, type of cataract, and surgical time, our findings indicate that patients were more likely to experience pain during second-eye surgery and reported more severe pain compared with first-eye surgery, which was consistent with the patients' chief complaint. The greater pain scores in second-eye surgery were correlated with lower preoperative anxiety scores. Moreover, our findings demonstrate the clinical significance of monitoring perioperative MAP and heart rate to evaluate and predict the levels of anxiety and perceived pain during cataract surgery under topical anesthesia.

Our study also showed that cataract patients were more likely to feel pain and reported significantly greater pain scores, during second-eye surgery compared with first-eye surgery. Our findings are consistent with those reported by Ursea et al. [1]. However, they only used the VAS pain scale to compare the difference in perceived pain between first-eye and second-eye surgery. Our results differ from the results of the study by Sharma et al. [2], in which the mean pain score was not significantly different between first-eye and second-eye surgery. However, the patients were orally sedated in the study by Sharma et al. [2] and cataract surgery was conducted by several different surgeons. In a study by Bardocci et al. [10], the patients did not receive oral sedation and all surgical procedures were performed by the same surgeon. However, they found no clear difference in the severity of pain between the first and second cataract extractions.

According to our results, the greater pain scores in second-eye surgery were likely to be correlated with the lower preoperative VAS anxiety scores compared with first-eye surgery, confirming the studies by Ursea et al. [1] and Foggitt [11]. From these results, we infer that patients who successfully underwent first-eye surgery may feel less anxiety before their second-eye surgery and may be more attentive to the level of comfort during cataract surgery, rather than how successful the surgery would be. Therefore, patients may perceive their pain to be significant during second-eye surgery.

Recent studies [12] have shown that objective measures, including blood pressure and heart rate in the perioperative period, may be correlated with subjective perceptions and might be influenced by the patient's anxiety and nervousness. Our study further investigated this issue. We evaluated whether perioperative objective measures, including blood pressure and heart rate, were correlated with each other or with perioperative pain and anxiety. To our knowledge, this is the first study to determine several objective measures and subjective measures of perceived pain in the perioperative period of cataract surgery. The inclusion of objective measures should reduce the possible bias associated with subjective measure. Bardocci et al. reported that 42% of females and 29% of males gave a final judgment of pain that was inconsistent with their reported VAS score for each procedure [10]. He suspected that some patients may not fully understand the scoring system. In our study, the perioperative blood pressure and heart rate were not significantly different between the two groups of patients. However, SBP, DBP, and MAP were significantly lower after cataract surgery than before surgery.

We also examined the correlations between anxiety scores and pain scores, and between subjective measures and objective measures. The results of these analyses confirmed our hypothesis described above.

In all of the enrolled patients, the preoperative anxiety scores were correlated with preoperative blood pressure and heart rate. Among patients who underwent second-eye surgery, the preoperative VAS anxiety scores were significantly correlated with postoperative WBS pain scores. Among patients who reported more severe pain in second-eye surgery than in first-eye surgery, the VAS pain scores were significantly and positively correlated with the differences between the intraoperative and postoperative values for DBP, MAP, and heart rate. Our study also showed that the greater perceived pain during second-eye surgery was correlated with lower anxiety levels before surgery, and the increases in MAP and heart rate during surgery may reflect the subjective pain level.

## 5. Conclusions

The results of the subjective (pain and anxiety) and objective (blood pressure and heart rate) measures in this study indicate that cataract patients were more sensitive to pain during second-eye surgery than during first-eye surgery. There was a subtle increase in the severity of pain in second-eye cataract surgery relative to first-eye surgery. This increase in pain appears to be associated with decreased preoperative anxiety scores. Preoperative blood pressure and heart rate could reflect the patient's anxiety level, while perioperative MAP and changes in heart rate could reflect the patient's perceived pain level. Therefore, despite considering subjective factors, such as preoperative anxiety monitoring, our findings indicate that perioperative MAP and changes in heart rate may be significant markers for preoperative anxiety and could predict the severity of perceived pain during cataract surgery.

## Supplementary Material

A questionnaire on anxiety and pain evaluation we designed, which combined the validated simplified State-Trait Anxiety Inventory, a visual analog scale for anxiety, a visual analog scale for pain and the Wong-Baker FACES Pain Rating Scale, is provided below with both English and Chinese versions.

## Figures and Tables

**Table 1:** Patient characteristics.

Parameter	First-eye surgery	Second-eye surgery	*P* value
Patients (*n*)	106	53	/
Mean age (y) ± SD	67 ± 13	69 ± 10	0.424
Gender (male/female)	40/66	22/31	0.646

SD, standard deviation.

**Table 2:** Differences between first- and second-eye cataract surgery.

Parameter	First-eye surgery (*n* = 106)	Second-eye surgery (*n* = 53)	*P* value
Pain rate (%)	35 (37/106)	87 (46/53)	<0.001
Median pain scores (range)			
VAS pain	1 (0, 8)	2 (0, 6)	0.001^∗^
Wong-Baker Faces Pain Rating Scale	2 (0, 6)	2 (0, 6)	0.003^∗^
Median anxiety scores (range)			
STAI	8 (6, 24)	7 (6, 24)	0.815
VAS anxiety	2 (0, 8)	1 (0, 8)	0.047^∗^
Median cataract (range)			
Nuclear	3 (2, 5)	3 (2, 5)	0.113
Cortical	0 (0, 1)	0 (0, 1)	0.564
Posterior subcapsular	0 (0, 1)	0 (0, 1)	0.576
Mean operating room time (min) ± SD	11 ± 3	10 ± 2	0.817
Mean phacoemulsification time (min) ± SD	0.9 ± 0.7	0.8 ± 0.6	0.842

Mean arterial pressure = systolic blood pressure × 1/3 + diastolic blood pressure × 2/3.

^∗^Significantly different between the two groups (two-tailed Wilcoxon test).

VAS, visual analog scale, STAI, State-Trait Anxiety Inventory; SD, standard deviation.

**Table 3:** Differences between two subgroups of patients who underwent bilateral sequential cataract surgery.

Parameter	More pain (*n* = 31)	Same or less pain (*n* = 22)	*P* value
Mean age (y) ± SD	71 ± 11	66 ± 8	0.073
Gender (male/female)	11/20	11/11	0.300
Median pain (range)			
VAS-pain	2 (1, 8)	1.5 (0, 4)	0.032^∗^
Wong-Baker Faces Pain Rating Scale	2 (0, 6)	2 (0, 6)	0.003^∗^
Median value of anxiety (range)			
STAI	6 (6, 24)	9.5 (6, 17)	0.566
VAS anxiety	1 (0, 8)	1.5 (0, 5)	0.293
Median cataract (range)			
Nuclear	3 (2, 3)	3 (2.5, 4)	0.260
Cortical	0 (0, 1)	0 (0, 1)	0.513
Posterior subcapsular	0 (0, 1)	0 (0, 1)	0.807
Mean operating room time (min) ± SD	10 ± 2	10 ± 4	0.831
Mean phacoemulsification time (min) ± SD	0.7 ± 0.3	1 ± 0.8	0.061
SBP/DBP (mmHg) ± SD			
Preoperative	147 ± 21/82 ± 11	145 ± 25/83 ± 14	0.760/0.815
Intraoperative	153 ± 23/85 ± 13	155 ± 22/89 ± 14	0.769/0.335
Postoperative	146 ± 23/79 ± 13	140 ± 22/78 ± 15	0.326/0.640
Mean arterial pressure (mmHg) ± SD			
Preoperative	104 ± 13	103 ± 17	0.959
Intraoperative	108 ± 15	110 ± 16	0.660
Postoperative	102 ± 15	99 ± 15	0.451
Heart rate (bpm) ± SD			
Preoperative	76 ± 13	73 ± 15	0.418
Intraoperative	76 ± 12	76 ± 14	0.960
Postoperative	75 ± 11	71 ± 17	0.203

Mean arterial pressure = systolic pressure × 1/3 + diastolic pressure × 2/3.

^∗^Significantly different between the two groups (two-tailed Wilcoxon test).

SD, standard deviation; VAS, visual analog scale, STAI, State-Trait Anxiety Inventory; SBP, systolic blood pressure; DBP, diastolic blood pressure.

## References

[1] Ursea R., Feng M. T., Zhou M., Lien V., Loeb R. (2011). Pain perception in sequential cataract surgery: comparison of first and second procedures. *Journal of Cataract and Refractive Surgery*.

[2] Sharma N. S., Ooi J.-L., Figueira E. C. (2008). Patient perceptions of second eye clear corneal cataract surgery using assisted topical anaesthesia. *Eye (London)*.

[3] Tan C. S. H., Fam H.-B., Heng W.-J., Lee H.-M., Saw S.-M., Eong K.-G. A. (2011). Analgesic effect of supplemental intracameral lidocaine during phacoemulsification under topical anaesthesia: a randomised controlled trial. *British Journal of Ophthalmology*.

[4] Ang C.-L., Au Eong K. G., Lee S. S. G., Chan S. P., Tan C. S. H. (2007). Patients' expectation and experience of visual sensations during phacoemulsification under topical anaesthesia. *Eye*.

[5] Nijkamp M. D., Kenens C. A., Dijker A. J. M., Ruiter R. A. C., Hiddema F., Nuijts R. M. M. A. (2004). Determinants of surgery related anxiety in cataract patients. *British Journal of Ophthalmology*.

[6] Boker A., Brownell L., Donen N. (2002). The Amsterdam preoperative anxiety and information scale provides a simple and reliable measure of preoperative anxiety. *Canadian Journal of Anesthesia*.

[7] Marteau T. M., Bekker H. (1992). The development of a six-item short-form of the state scale of the Spielberger State-Trait Anxiety Inventory (STAI). *British Journal of Clinical Psychology*.

[8] Millar K., Jelicic M., Bonke B., Asbury A. J. (1995). Assessment of preoperative anxiety: comparison of measures in patients awaiting surgery for breast cancer. *British Journal of Anaesthesia*.

[9] Tomlinson D., von Baeyer C. L., Stinson J. N., Sung L. (2010). A systematic review of faces scales for the self-report of pain intensity in children. *Pediatrics*.

[10] Bardocci A., Ciucci F., Lofoco G., Perdicaro S., Lischetti A. (2011). Pain during second eye cataract surgery under topical anesthesia: an intraindividual study. *Graefe's Archive for Clinical and Experimental Ophthalmology*.

[11] Foggitt P. S. (2001). Anxiety in cataract surgery: pilot study. *Journal of Cataract and Refractive Surgery*.

[12] Deschaumes C., Devoize L., Sudrat Y., Baudet-Pommel M., Dualé C., Dallel R. (2014). The relationship between resting arterial blood pressure and oral postsurgical pain. *Clinical Oral Investigations*.

