# Supplementary material for: Perceived Pain during Cataract Surgery with Topical Anesthesia: A Comparison between First-Eye and Second-Eye Surgery
Source: J Ophthalmol. 2015 May 4;2015:383456. doi: 10.1155/2015/383456 (PMC4434194; doi:10.1155/2015/383456)
Supplement: Supplementary file 1 — A questionnaire on anxiety and pain evaluation we designed, which combined the validated simplified State-Trait Anxiety Inventory, a visual analog scale for anxiety, a visual analog scale for pain and the Wong-Baker FACES Pain Rating Scale, is provided below with both English and Chinese versions. [file 383456.f1.pdf]

Supplementary materials: Questionnaire 1 (English Version)
Number___________

Eye and ENT Hospital, Fudan University
Anxiety questionnaire of cataract surgery

Name______ Gender_____ Age_____ 1st/2nd-eye surgery Date________

Simplified STAI questionnaire

	**Not at all**	**Somewhat**	**Moderately**	**Very much**
I feel calm	4	3	2	1
I am tense	1	2	3	4
I feel upset	1	2	3	4
I am relaxed	4	3	2	1
I feel content	4	3	2	1
I am worried	1	2	3	4

Total points ___________

10-point visual analog scale (VAS)

+-----+----+-----+-----+-----+-----+-----+-----+-----+------+
0 1 2 3 4 5 6 7 8 9 10
Not anxious at all The most anxious I can imagine

Objective measures:
Blood pressure:
Pre_____ /_____mmHg, Intra_____ /_____mmHg, Post_____ /_____mmHg

Heart rate:
Pre____________bpm, Intra____________bpm, Post____________bpm

Supplementary materials: Questionnaire 1 (Chinese Version)

复旦大学附属眼耳鼻喉科医院
白内障手术焦虑情况表

姓名____________ 性别_____ 年龄_____ 第___眼手术 日期________

一、 SATI 简化问卷

	**没有**	**有一些**	**中等程度**	**很重**
我觉得心情很**平静**	4	3	2	1
我觉得**紧张**	1	2	3	4
我觉得**心烦意乱**	1	2	3	4
我觉得很**放松**	4	3	2	1
我感到**心满意足**	4	3	2	1
我很**担心**	1	2	3	4

总分：___________

二、10 分视觉量表（VAS）

我觉得我的紧张程度：

+-----+-----+-----+-----+-----+-----+-----+-----+-----+-----+

0 1 2 3 4 5 6 7 8 9 10

客观指标：

血压：术前_____ /_____mmHg 术中_____ /_____mmHg 术毕_____ /_____mmHg

心率：术前____________次/分 术中____________次/分 术毕____________次/分

Supplementary materials: Questionnaire 2 (English Version)

Eye and ENT Hospital, Fudan University
Pain Questionnaire of cataract surgery

Surgical Information

Visual acuity before surgery _________

Anesthetic method: topical / retrobulbar

Cataract type: Age-related / Secondary (Cause: __________)

Cataract degree: N (nuclear)_____C (cortical)_____P (subcapsular)_____

Operating room time: ______minutes_______seconds

Phacoemulsification time: ______minutes_______seconds

Pain Scale

Please select your pain level during surgery (mark the appropriate numbers):

1. 10-point visual analog scale

+–––––+––––+–––––+–––––+–––––+–––––+–––––+–––––+–––––+––––––+

0 1 2 3 4 5 6 7 8 9 10

Not painful at all The most pain I can imagine

2. Wong–Baker FACES pain rating scale

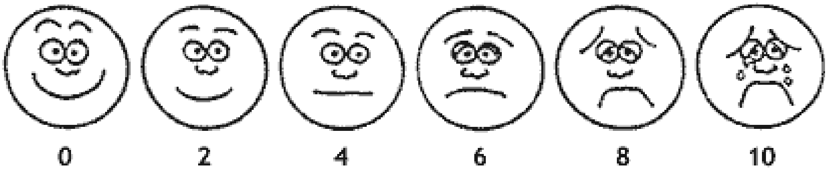

0 = Not painful at all

2 = A little bit painful

4 = Somewhat painful

6 = Obviously painful

8 = Very painful

10 = Extremely painful

3. Additional question for patients undergoing second-eye surgery:
Compared with my first-eye surgery,

I had more pain during the first procedure ( )

I had more pain during the second procedure ( )

I experienced the same pain during both procedures ( )

I can't remember ( )

Supplementary materials: Questionnaire 2 (Chinese Version)

复旦大学附属眼耳鼻喉科医院
白内障手术疼痛情况表

一、手术情况

术前视力 _________

麻醉方式 表麻 / 球后

白内障类型 老年性 / 并发性（病因：__________）

白内障分级 N（核）_____C（皮质）_____P（后囊下）_____

手术时间 ______分_______秒

超声时间 ______分_______秒

二、疼痛程度

我觉得手术中疼痛程度（在相应数字上画圈）：

1. 10 分表

+-----+-----+-----+-----+-----+-----+-----+-----+-----+-----+

0 1 2 3 4 5 6 7 8 9 10

不痛 一生中感到的最痛

2. Wong-Baker 笑脸表

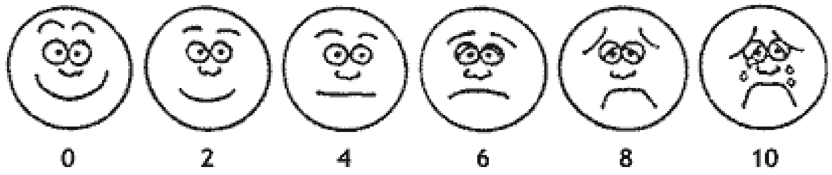

不痛 有一点痛 更痛一点 明显疼痛 很痛 特别痛苦

3. 第二只眼手术询问：与上次手术相比哪次手术更痛

上次更痛 （ ）

本次更痛 （ ）

两次差不多 （ ）

记不清 （ ）
